# The shadows of waiting and care: on discourses of waiting in the history of the British National Health Service.

**DOI:** 10.12688/wellcomeopenres.18913.1

**Published:** 2023-02-13

**Authors:** Shaul Bar-Haim, Lisa Baraitser, Martin D. Moore

**Affiliations:** 1 Department of Sociology, University of Essex, Colchester, UK; 2 Psychosocial Studies, Birkbeck University of London, London, London, WC1N 7HX, UK; 3 Wellcome Centre for Cultures and Environments of Health, University of Exeter, Exeter, UK

**Keywords:** NHS Historiography, Waiting, Waiting Lists, Waiting Times, Healthcare, Health Economics

## Abstract

Waiting is at the centre of experiences and practices of healthcare. However, we know very little about the relationship between the subjective experiences of patients who wait in and for care, health practitioners who ‘prescribe’ and manage waiting, and how this relates to broader cultural meanings of waiting. Waiting features heavily in the sociological, managerial, historical and health economics literatures that investigate UK healthcare, but the focus has been on service provision and quality, with waiting (including waiting lists and waiting times) drawn on as a key marker to test the efficiency and affordability of the NHS. In this article, we consider the historical contours of this framing of waiting, and ask what has been lost or occluded through its development. To do so, we review the available discourses in the existing literature on the NHS through a series of ‘snapshots’ or key moments in its history. Through its negative imprint, we argue that what shadows these discourses is the idea of waiting and care as phenomenological temporal experiences, and time as a practice of care. In response, we begin to trace the intellectual and historical resources available for alternative histories of waiting – materials that might enable scholars to reconstruct some of the complex temporalities of care marginalized in existing accounts of waiting, and which could help reframe both future historical accounts and contemporary debates about waiting in the NHS.

## Introduction

News headlines in the British press have been dominated in recent years by a mounting sense of ‘crisis’ across the health and social care system. During 2018, as the National Health Service turned 70, its ‘birthday’ occasioned a national debate about the ‘health’ of the health service and whether it was now in ‘permanent crisis.’ ^1^ The Covid-19 global pandemic that was underway in the UK by March 2020 deepened the sense of an already over-stretched service at the edge of breaking point. By 2022 waiting lists and waiting times reached alarming proportions, and an early report by the National Audit Office described a possible 12 million-strong waiting list by March 2025, driven by the re-emergence of between 7.6 and 9.1 million ‘missing’ referrals for cancer care during the pandemic ( NAO, 2021). Whilst the sentiment of crisis is conveyed by figurations of collapse – with the NHS described as ‘dying’, ‘at breaking point’, ‘melting down’ and ‘unable to cope’ – the evidence of crisis is often presented in terms of long waiting times. ^2^ Whether the wait is for an ambulance, to be seen in Accident and Emergency, for an appointment with a General Practitioner (GP), for mental health treatment, or for consultant-led elective care, increased waiting times are produced as evidence of service failure.

Yet waiting as a form of temporal extension is at the core of experiences and practices of healthcare. Often caught between the push-pull of both urgency and chronicity, patients, health professionals, support staff and NHS managers, constantly find themselves prescribing, experiencing, negotiating and managing waiting. This is not just a result of a service under pressure, but because waiting has a function in diagnosis, treatment, recovery, remission, and dying. Patients, too, individually and collectively navigate periods of illness and the interstitial time between healthcare activities in ways that can confer meaning or make the unbearable endurable. Even to use the word ‘patient’ is to recognize, at least at an etymological level, the patience, endurance, suffering, and resilience of healthcare users and their carers as they wait and see what giving time to a situation brings. ^3^

Although the current problems of waiting in the NHS are greatly disconcerting, the filtering of waiting in and for healthcare almost exclusively through lenses of waiting lists, waiting times, and service crisis dislodges broader understandings of waiting in relation to care. To some extent waiting as a historically situated, phenomenological experience and as an element of treatment are so embedded in everyday practices of care that they can be easily overlooked. They form, to borrow from Joe Moran, part of healthcare’s ‘infra-ordinary’ – aspects of daily life ‘that often go unremarked upon because they become social habits rather than individual idiosyncrasies’ ( Moran, 2007: 3). However, they can also be particularly difficult to discuss during periods in which unwanted and unnecessary waiting increases, and becomes intolerable or even dangerous. We do not deny that waiting in and for health and social care can be painful, anxiety-provoking and, in some instances, deleterious to health. But while we support calls to reduce unnecessary waiting times, we are nevertheless concerned that the crucial role that waiting plays in care and treatment requires better conceptualization. Waiting as an essential part of healthcare needs to be reconnected to wider debates about changing perceptions, experiences, and social organizations of time so that the value of waiting as a form of careful and caring attention does not get lost ( Salisbury *et al.*, 2023).

As broader questions of temporality – of waiting as an experience, a quality of relating, or a practice of careful attention rather than an absence of action – are difficult to broach in the present, this article turns to historiography to see whether we can shift contemporary discussion by locating such explorations of waiting in the past. ^4^ We investigate discourses of waiting within the history of the NHS as they emerge in some of the key historical, sociological, and economic literatures over the last 70 years, literatures that in part act as the NHS’s internal critique. However, through three predominantly historiographic snapshots of the 1950s bed crisis, 1960s general practice, and the managerial culture of the NHS in the 1980s and 1990s, we argue that the lacunae that manifest around waiting in contemporary conditions are also encountered historically. Waiting lists and waiting times may only have become the dominant frames for discussing waiting within the NHS after the 1970s ( Sheard, 2018) but we show that even prior to this point waiting often only breached the surface of political and academic discourse in relation to questions of resource management and temporal economy. The dominance of these framings of waiting in historic policy and academic literatures has, moreover, been little altered by an NHS historiography that, in the words of Snow and Whitecross, has only recently ‘become interested in dimensions of NHS history that stretch beyond its political, policy, clinical and administrative characteristics [and] dimensions’ (2022: 405). Like scholars in sociology, health economics, public health policy and health management, historians have generally only attended to waiting as it emerges within a politics of healthcare. Even landmark deconstructions of how policymakers came to prioritize reduced hospital waiting ( Sheard, 2018) or accelerated general practice access ( Simpson *et al.*, 2018) have, by necessity, fixed their analytic gaze upon particular political expressions of temporal suspension and elongation. ^5^

Faced with the same challenges as research that investigates ‘silences’ and ‘absences’ within an archive, we here trace the more visible twin of waiting as an experience or practice of care, namely the research that charts waiting as a measure of service provision and debates about how to reduce and eliminate waiting in health services. ^6^ By historicizing these narratives of waiting and by tracing the negative imprint of care shadowing these extant histories, we want to set the ground for developing alternative historical sensibilities and perspectives: ones that might enable future work to look beyond ingrained constructions of waiting within narratives of crisis and structures of service management. By highlighting how historic and historiographic accounts of waiting have often occluded thinking beyond the time politics of healthcare – the organization, management and technologies of time and its governance – we aim to give form to a silence which threads its way through the history of the NHS, even if we cannot fill it directly. And by considering what materials and frameworks are available for recuperating broader histories of waiting, we hope that this task will be taken up by a wider set of scholars: not just historians, but sociologists, anthropologists and researchers from other social sciences and humanities fields hitherto surprisingly quiet on the issue. ^7^ Given the centrality of different forms of waiting to contemporary healthcare and politics, and to cultural histories of the NHS, this work is imperative. Current framings of NHS waiting beg innumerable important questions: how, for instance, do particular embodied subjects experience waiting within the same systems or encounters? How have wider historical, cultural and social organizations of time impacted subjects’ experiences of waiting in and for healthcare? How might we capture subjective forms of waiting as they intersect with personal and cultural histories? And how has power manifested within a social history of waiting, shaping who waits, and has waited, for whom, for how long and why? Enabling future work to respond to such questions, we suggest, is essential for understanding waiting within the NHS as a historically significant, and historically situated, set of practices and experiences ( Hage, 2009; Moran, 2005).

To begin addressing the historiographic problem of waiting in the NHS, then, this article will be divided into five sections. Part one briefly connects what follows to a broader literature on the social and material life of time, to underline the historically contingent nature of time within healthcare. From here, parts two, three and four offer snapshots of key points of transformation in NHS temporal frames. We examine the ‘bed crisis’ of the 1940s and 1950s, in which the hospital bed – and especially the ‘pay bed’ – becomes entangled in new discourses around acute and chronic illness, medical and non-medical need, and the deserving and non-deserving in a health service envisioned as free at the point of entry for all. Through these entanglements, new categories of waiting and exclusion were produced with repercussions for later developments in the NHS. We then outline the consolidation of a new temporal economy in general practice, which was simultaneously bound up with the creation of new forms of patient biography and chronicity and provided the basis for novel managerial cultures after the 1970s. This leads us to take up the history of managerialized waiting over the 1980s and 1990s, exploring how subjective experiences of waiting and collective movements for health equity were marginalized within growing debates about the relative importance of waiting lists and waiting times in governing waiting. Finally, part five offers some concluding thoughts on alternative resources available for exploring the current shadows of waiting and care, suggesting that developing more eclectic archives of waiting and drawing on novel projects for exploring cultural histories of the health service might enable new histories of waiting to be told.

## The social and material life of time

In thinking about waiting times literatures historically, it is important to acknowledge that we are building on a rich body of work on the social and material life of time and its historical and cultural construction. ^8^ Temporalities studies scholars have, for instance, long rooted the generalization of clock time across Europe within sixteenth-century wage labour and the subsequent development of industrial market relations, whilst also highlighting its foundational role in imperial expansion and colonization ( Nanni, 2012). Clock time produced a temporal imaginary in which time was conceived of as neutral, constant and measurable, a medium through which we move in a linear and irreversible direction ( Hutchings, 2008), interrupting and overriding local and Indigenous relations to time in many parts of the industrial and colonized world. Historically such transformations met concerted resistance ( Ogle, 2015; Thompson, 1967) and produced intense cultural reactions ( Kern, 1983). Moreover, over the past four decades, scholars writing from Black, crip, Indigenous, feminist, post-colonial and queer perspectives have consistently critiqued the abstract, productionist and progressivist temporal frames entangled with the clock for their imposition of temporal norms that underpin oppression, marginalization and historic forms of racialized economic extraction. ^9^

For our purposes, this work underlines that the ways we live and conceptualize time are not static, either historically or geopolitically. Time is framed as a socially produced narrative, or as a category of experience that itself has a ‘social life’, disrupting the idea that time is a blank backdrop against which history unfolds. Furthermore, scholars have a long history of mobilising such framings in the study of healthcare, exploring it as a particularly resonant site for understanding the social production and organization of time. ^10^ Within healthcare, questions of chronicity and urgency in relation to illness and its treatment, the time of living and dying, of rhythm and pace, crisis and remission and the timeliness of medical intervention, play out alongside constructions of time that challenge or enact particular forms of relationality, including relations between healthcare practitioners, their patients and the NHS as an institution. Indeed, inside a system of healthcare as complex as the NHS this relationality often assumes modes of waiting, whether expected, prescribed or unanticipated ( Day, 2015).

Of course, as David Armstrong (1985) makes clear, British medicine’s temporal constructions and relationalities have proven as historically contingent as those elsewhere. In the following three sections of the paper, therefore, we offer a series of snapshots that trace historical trends relating to the creation of ‘waiting’ as a major priority for the public system, but which simultaneously obfuscated patient and practitioner experiences of waiting in and for healthcare.

## Snapshot one: the ‘bed crisis’

The most important – almost mythical – element of the Bevanite ethos for the NHS was the mantra of providing a free medical service to all citizens and subjects, funded by general taxation. Yet, as is well documented, over the first decade the available funding did not fulfil expectations of a modern national system and the Service struggled to respond effectively to the multiple and diverse needs of patients. Troubles emerged almost immediately with a major gap between actual expenditure and that predicted in legislative documents for the health services, creating a financial and political crisis ( Cutler, 2003; Webster, 1988). Though the findings of the Guillebaud committee in 1956 set the NHS on a sounder political footing ( Webster, 2002), organizational complexities and pent-up demand made the nascent Service difficult to manage ( Waller, 1996), even with the emergence of notable innovations in training and staffing ( Snow, 2013). ^11^ As historical accounts suggest, these intractable issues manifested within hospital waiting lists, drawing early political attention ( Waller, 1996).

Without significant new investment in staffing or infrastructure, a subsequent shortage in beds increased pressure to reduce the time patients spent in hospital (254-55). ^12^ This included the reassessment of former definitions of chronic illness, poverty, disability and older age. Prior to the establishment of the welfare state, many who did not have acute medical need nevertheless stayed in hospital for long periods, sometimes for years ( Lowe & McKeown, 1950). ^13^ The bed shortage led to the removal of older people, for instance, into long-stay residencies and halfway houses. Though there were arguments that these provided better care, they were ill-supported financially and many were inadequate. Removing older patients from hospital was also part of an ongoing debate that had been brewing since the interwar years about whether those in older age constituted a social or medical ‘problem’. Although trans-Atlantic developments were challenging received views on ageing ( Armstrong, 2014) and doctors were willing to admit the elderly into the realm of curative medicine from which they had long been excluded ( Martin, 1995), it took much longer to distinguish between chronic illness and older age, categories of experience that were perceived by many practitioners as synonymous (249–250), and between those in need of medical care and other complex social, financial and psychological forms of support. Through these reconfigurations of *clinical* desert and need, pressures to make beds available to some patients – even if they had to wait – resulted in denying such claims made by and for others. Whilst the development of geriatric medicine as a profession accelerated from the 1940s onwards ( Denham, 2006; Martin, 1995; Pickard, 2010), the elderly, poor, disabled and chronically ill were effectively removed from long-term hospital settings.

Clinical criteria alone, of course, did not determine access to British hospital beds and debates about waiting lists and clinical efficiency soon became entangled with questions of private practice. Despite the NHS being built on hospital nationalization, ‘pay beds’ had existed within NHS institutions since 1948. They formed part of the price Aneurin Bevan and the Labour Party paid to retain specialist doctors within the public system and thus pockets of private provision were structural to the NHS from its inception ( Klein, 2013; Webster, 1988). Their inclusion within a service ostensibly founded on need, however, proved controversial ( Williamson, 2015). Pay beds had to be profitable, otherwise the public would end up subsidizing the wealthy and there were ongoing efforts to publicly legitimize what was perceived by many as ‘jumping the queue where sickness is concerned’ (584). Indeed, pay beds introduced non-clinical notions of access and ‘timeliness’ in relation to care, allowing patients to pay not just for care by a consultant of their choice, but crucially to buy healthcare at a time convenient for them (577). These issues were hotly debated throughout the 1950s and 1960s, especially when it became clear that many beds were kept empty for *potential* paying patients, structuring the wait for patients in need of public resources. ^14^ During the 1950s, the Labour Party demanded that pay beds be abolished. However, when returning to power in 1964 another promise – to cancel prescription charges – assumed political priority and legitimated retaining pay bed income. Instead, to avoid conflict with consultants, the government cut the number of pay beds within the Service and changed the authorization system to increase their occupation. 

Notions of ‘timely treatment’ and questions about ‘who waits’ therefore emerged early in the history of the NHS, through debates around what constituted a ‘chronic’ (as opposed to an ‘acute’) problem and conflicts around pay beds intersecting with broader discourses of bed crisis in the 1950s. The construction of ‘waiting’ as a category of experience for some and not for others was thereby entangled in this period with contested delineations of *which* embodied subjects were allowed to occupy beds (or disembodied subjects, in the case of beds kept empty for potentially paying patients), *when* such subjects were allowed to occupy beds and *who* waited for a bed as a result. In other words, only those deemed to be in *medical* need (increasingly narrowly defined), in hospital, came to be counted as ‘waiters’ for the public system. To wait meant to have a claim on the specialized and institutional provisions of the health service, but at the expense of those discounted from waiting at all. These ‘shadow’ waiters – disproportionately the poor, the chronically ill, those living with disabilities and older individuals – were structurally removed from the early history of NHS waiting and their experiences of being disallowed from waiting for and in hospital settings remain occluded. Instead, they found themselves gradually displaced into the less valued and less resourced sectors of NHS community care and general practice. These shadow waiters were not passive victims in a broader story of post-war welfare ( Bradley, 2019; Hampton, 2017; Thane, 2011), but here their struggle was to become a waiter at all, in the face of structural pressures to delimit who was eligible to wait for healthcare.

## Snapshot two: General Practice and the new economy of time

As historians and sociologists have outlined, general practice was itself consolidating transformations in its conceptualizations of time over the 1960s and 1970s. New technologies and concepts of biography, risk and time management contributed to a reconstruction of chronicity within community care, just as a new economy of time began to dominate primary care during these decades. As demands for consultation increased – and as new notions of ‘illness’ stretched and folded past, present and future in novel ways – efforts to measure and regulate time as a scarce resource became more prominent than in general practice’s earliest years. These were shifts that repositioned those ‘shadow waiters’ in relation to the NHS once again and fed into broader managerial transformations at the century’s end.

Changes in general practice, of course, occurred in the context of broader organizational adjustments across the NHS, starting with capital investments announced under the Hospital Plan in 1962 ( Gorsky, 2008; O'Hara, 2007: 179-190). A new family doctor contract was agreed in 1966, flanked by various state committees to investigate the separate parts of the NHS and its co-ordination ( Rivett, 1998), and the NHS itself was restructured in 1974 as part of a drive to rationalize its administration. The restructure devolved more power to local and regional authorities and unified hospital and local government services, ending the previous division of NHS administration into hospital services, family health services and local authority provision ( Begley & Sheard, 2019; Ham, 2009: 23-28; Webster, 1996). However, these changes were not simply concerned with efficiency. Some commentators and practitioners hoped that new arrangements could re-balance the NHS’s focus, to counteract the ways in which technologically intensive hospital-based services for acute illness absorbed resources and attention, to the detriment of community-based primary care and care of the chronically ill ( Battistella & Chester, 1973: 493).

One reading, then, of health policy between the 1960s and mid-1970s is of an attempt to shift towards more community-based health services. This new focus was brought about by a recognition of the multiple temporalities of health and illness and the need for a service that could respond more flexibly to the acute, the chronic, and everything in between. Notably, general practitioners influenced reforms in this direction. Their collective action shaped the 1966 contract ( Lewis, 1998; Rivett, 1998) and secured reforms to referral and laboratory testing mechanisms that enabled greater community-based diagnosis and treatment ( Bosanquet & Salisbury, 1998). Moreover, new arrangements were intended to develop practice teams that could more efficiently meet the complex needs of patients. Changes to remuneration and financial support schemes, for instance, fostered the development of group practices and the hiring of practice administrative staff, whilst closer working ties with nursing and other community care staff were supported by local authority ‘attachment schemes’ ( Bosanquet & Salisbury, 1998).

A constitutive element of these changes was investment in the use of appointments systems, which gradually replaced ‘open surgery’ arrangements run on a ‘first come-first served’ basis ( Bosanquet & Salisbury, 1998: 55-6). In 1964 only around 15 per cent of GPs used an appointment scheme for their surgery hours, whereas by 1977 this had risen to 75 per cent. The politics and motivations for creating appointments systems were complex ( Moore, 2022). But, crucially, new systems restored a sense of temporal autonomy for GPs, whilst also giving doctors greater control and flexibility in organizing their timetable and the capacity to order, smooth and slow the demands on their time made in any given clinical session ( Bosanquet & Salisbury, 1998: 56; Moore, 2022). They also potentially offered GPs a more systematic way to maintain forms of temporalized surveillance and ‘watchful waiting’: they eventually became essential to structured follow-up of recognized long-term patients such as patients with diabetes ( Moore, 2019) and they created more time for doctors to longitudinally observe patients before deciding whether referral was necessary. Loose invitations to patients to return in a week or month, for example, could now be formalized calendrically and bureaucratically.

On the one hand, therefore, such systems supported the creation of extended, more defined periods of waiting outside the surgery ( Moore, 2022), whilst contributing to new understandings and practices of time by structuring care for the ‘not-yet-urgent’. Indeed, this blurring of temporal lines – remaking distinctions between the ‘urgent’ and ‘not-urgent’ in GPs’ offices and consulting rooms – contributed to a broader reworking of notions of the ‘chronic’ within British medicine and public health ( Berridge, 2007; Weisz, 2014). Together with the increasing importance of medical records to the longitudinal management of patients in general practice, inscription devices like appointments systems fostered new understandings of illness in which ‘time became concatenated’ ( Armstrong, 1985: 663). Biographical, biological, social and organizational temporalities were reformatted within novel notions of psychosocial illness ( Bar-Haim, 2018; Hayward, 2014) where the ‘past informed and invaded the present’ ( Armstrong, 1985: 663), as well as in novel forms of primary care ‘risk’ management ( Moore, 2019), in which past and present were re-read to manage potential pathological manifestations in the future ( Armstrong, 1995). These technologies of general practice – the appointments system and the medical record – were thus vital actors in creating a chronicity that intervened in the grammar of ‘crisis’. They helped to produce a time ‘in between’ that was distinct from the duration of an illness, and with it a new ‘chronic subject’. It was a subject who, in some senses, was always in a state of waiting.

On the other hand, appointments systems, with their segmentation, standardization and regimentation of the flow of consultation time, embodied a growing objectification and quantification of time within general practice. Having mixed contract and fee-for-service work since the nineteenth century, GPs had long conceived of time as a resource whose prudent use could generate capital rewards ( Digby, 1999). Similarly, feelings of ‘time pressure’ and rush were common among GPs in the earliest years of the NHS ( Moore, 2022). Some practitioners even responded to such experiences by engaging health services research ( Armstrong, 1985), work that revealed stark variations in average consultation times, hours of work, and workload by season, area, and more ( Fry, 1952; Taylor, 1954). However, during the 1960s and 1970s, research into GP workload and consultation times became increasingly common ( Buchan & Richardson, 1973; Eimerl & Pearson, 1966). But it was also structured by political and professional projects promoting greater GP self-reflection ( Osborne, 1993) and technocratic service management ( Moore, 2019), as well as rising per-capita rates of consultation and shifting patterns of medical labour within team-based surgeries.

Indeed, though investment in GP recruitment had lowered average patient registrations per doctor from 2,360 in 1966 to 1,812 by 1991 ( Bosanquet & Salisbury, 1998), potential time ‘savings’ were eradicated by growing patient demands and expanded intervention in new sets of chronic problems. ^15^ Primary care resources, in other words, were consistently outstripped and doctors found themselves frequently debating the need to extend the time given to each patient, which was often not more than six minutes ( Balint & Norell, 1973; Horobin & McIntosh, 1983: 315). Discourses of efficiency subsequently entered a self-intensifying loop and GPs consistently explored ways in which they might create time by reallocating it from elsewhere: from the use of appointment systems to deputing responsibilities and reducing home and follow-up visits ( Bosanquet & Salisbury, 1998).

We could argue, then, that the major innovation in the appointment system was not a new way of rationing time, but the solidification of an epistemological shift in the medical profession itself and in patients’ relationship with it. Though NHS GPs had always felt the weight of the waiting room within their consultations, the appointment system more tightly quantified the formal offer and expected duration of patient interaction. As part of a broader response to new pressures, patterns and perceptions of practice, appointments made it more difficult for doctors to simply ask ‘what can I do for this patient?’. Instead, the urge to ask ‘what can I do for this patient in the next six to 10 minutes’ became more difficult to resist. A shortage of time became more firmly lodged as a perceptual structural feature of general practice, independent of how individual practitioners and their patients experienced it in the everyday of individual consultations ( Horobin & McIntosh, 1983: 328). It was a perceptual feature that could easily mitigate against poorer, older and socially marginalized patients who were more likely to be on crowded lists with worse health and whose chronic problems (especially after being excluded from the hospital) were being reformatted increasingly into disaggregated and bureaucratic forms of management. Moreover, embodied in new technologies of time management, this perception of structural constraint in which time is measurable and specific in its aims, and is constantly lacking, under pressure or running out, laid the ground for the emergence of the new managerial discourse in health policy in the 1980s. This was not necessarily a ‘tyranny of time’ (313), but a more stringent discourse of time’s ‘economy’ that characterized many aspects of the late twentieth-century NHS and which continues to dictate large parts of the medical profession as a whole.

## Snapshot three: consumerist and managerial turns

Although productionist discourses of time’s economy are closely associated with the growth of executive management within the NHS, historical and sociological examinations underline that these discourses did not emerge *de novo* in the 1980s. Nor were they exclusive to hospital settings. Nonetheless, the decades after 1970 did provide a turning point in the way that waiting and efficiency became conjoined, particularly within secondary and tertiary care settings. Between the 1970s and early 2000s, waiting times and lists became scrutinized much more intensively by diverse political actors and became subject to considerably more centralized governance and scholarly attention ( Sheard, 2018). What was edged out by this intensifying drive to reduce waiting was any sense of how waiting and care might intersect – whether in a phenomenological sense or as part of a collective dedication to equity.

This failure to incorporate patients’ perspectives was ironic given that a policy focus on waiting times and lists was supported by organized patients’ bodies. As Alex Mold (2015) has explored, the 1960s and 1970s saw the proliferation of grass-roots groups and lobbies *of* patients *for* patients, part of a broader ‘consumerist’ movement in British politics ( O’Hara, 2013). Whilst these groups employed languages of choice which could be identified with the making of an individualized ‘patient-consumer’ ( Mold, 2011), they nevertheless aimed to represent the interests and subjective perspectives of patients in their collective engagement with health services. Significantly, issues relating to waiting quickly became a point of interest. ^16^ The College of Health, for instance, published for the first time its *Guide to Hospital Waiting Lists* in 1984. Unsurprisingly, in line with consumer groups’ emphasis on collective patient experiences and priorities, the College’s work focused less on service ‘performance’ and more on questions of equity. Their report provided not only troubling figures on the number of people waiting to be treated in hospitals and how long they waited, but also data about inequalities in access to treatment in different geographical locations ( Mold, 2015; Sheard 2018). However, alerted to such variations, state schemes for computerization and waiting list reduction continued policies of short-term investment to reduce list sizes ( Sheard, 2018). Experiences of waiting that were gathered as part of a fight for justice, equality and voice ( Mold, 2015), became twisted into a discourse of ‘proficiency’ and mobilized within ideological tussles about how to produce a more efficient and accountable NHS. The idea of waiting as an uneven but perhaps necessary experience, one that could potentially be shared out more equitably, was subsumed by a now hegemonic discourse of efficiency, with waiting understood as something to be eliminated at all costs.

As Sheard (2018) has noted, then, the growing influence of neoliberal political economy on health services policy was a constitutive element of more managerialized approaches to waiting during these decades. Such approaches were connected particularly to the introduction of new executive and professional management structures following the 1983 Griffiths Inquiry ( Gorsky, 2013). The Inquiry made numerous suggestions for reform, most prominently the employment of new types of managers in the Service, who would be more committed to running the health system ‘successfully’ in financial and organizational terms rather than from the perspective of the profession ( Gorsky, 2013). As Gorsky (2013) notes, such suggestions might not have been successful in the short term. They were, however, consolidated over time, in tandem with other aspects of neoliberal innovations in health service management such as devolved budgeting, the use of performance benchmarking and new forms of quality assurance ( Moore, 2019). Within this regime, waiting lists and waiting times were transformed into ‘quality’ indicators and markers of service ‘efficiency’, subject to managerial and state intervention.

This quantification and managerialization of waiting was, moreover, sustained by the growth of health economics over the 1960s and the integration of health economists into service management in the late 1970s ( Sheard, 2018). During this time, health economists began to differentiate between waiting *lists* and *times* and debate how best to measure waiting times themselves ( Culyer & Cullis, 1976). An early paper by Jones and McCarthy (1974), for instance, suggested that political clamour over waiting lists was only partly justified, as increases were specific to several surgical specialties. The authors drew attention to how data on waiting lists was gathered, stating that ‘what really matters to patients is the total waiting time between the appointment being made for them to attend at the hospital and the time when they receive definitive treatment, either as an outpatient or inpatient’ (35). Defending the system, yet using a nascent language about ‘what really matters to patients’, the authors suggested that ‘the first essential is to define what can be regarded as a reasonable average waiting time to ensure best use of present resources’ (36). These parameters – a reasonable average waiting time, methods for its measurement and the discourse of finite present resources – would stand at the heart of debates in the following decades.

We can see how patient experiences that drove the College of Health’s data collection slipped from view in a health services research literature that saw its task as responding to the challenges of measurement and management rather than equity and patient voice. Even as the focus on waiting lists reappeared in later years with a new demand to know more socio-economic details about patients who waited ( Davidge *et al.*, 1987), health economists paid little attention to the experiences of patients themselves or to the concerns of fairness underpinning the College’s work. Rather, they developed methods for data collection and analysis to inform different financial and econometric models about the cost-effectiveness of lists, their length, their impact on the economy and the economic effect of NHS users. ^17^ Scholars debated, for instance, how to properly cost the consequences of delay to both the patient and ‘the system’, with considerations of the ‘waiter’s’ health and emotional state converted into factors to be calculated and measured ( Lindsay & Feigenbaum, 1984; Propper, 1990:193). The tussle about the model’s effectiveness, in other words, was primarily economic.

Social science researchers and political figures continued to debate the relative merits of prioritizing waiting times or waiting lists as technologies of temporal management into the 1990s ( Klein *et al.*, 1995; Oliver, 2005; Pope, 1992), but rarely were the broader features of waiting for patients and practitioners considered in such discussions. Moreover, the development of both technologies within health economics worked hand in hand with the intensification of discourses of privatization during this period, whereby waiting as experience, as a source of meaning and as a form of care, became subsumed by the problems of too many waiting, and waiting too long.

Indeed, both the measurement of waiting and the acceptance of privatization came to span the political spectrum across the later 1980s and early 1990s, as waiting was once again closely connected with discussions about private provision ( Cullis & Jones, 1985; Propper, 1988; Yuen, 1991). These associations assumed multiple forms. On the one hand, studies suggested that most patients opted for private healthcare precisely to avoid waiting for treatment ( Higgins & Wiles, 1992) and there was also evidence that those who waited longer – often under the NHS – had poorer prognoses ( Marber *et al.*, 1991). On the other, critics of privatization argued that there was a close link between waiting and the growth of private services in areas with the longest waiting lists ( Richmond, 1996). Some claimed that doctors were manipulating waiting lists to enlarge their private income; others preferred to talk in terms of the indirect influence doctors may have had over the growth of lists ( McAvinchey & Yannopoulos, 1993). All such criticism met with robust rebuttal from neoliberal policymakers, with management discourse providing new tools to argue that there was no connection between waiting lists and public resources ( Frankel, 1989). Waiting lists, they suggested, were solely the outcome of non-professional management that required a stronger steer.

Ultimately, this steer was forthcoming under the New Labour administrations of the early 2000s, as the reduction of both waiting times and waiting lists became a focus of cross-party, centralized forms of New Public Management. ^18^ Under what became unofficially dubbed the ‘Targets and Terror’ scheme, the Department of Health committed to cutting the maximum wait for inpatient treatment from 18 months to six months, *via* staged reductions, in the year 2000. ( Propper *et al.*, 2008). Crucially, in a highly controversial move to achieve these goals, Labour governments used published league tables, sanctions against managers who missed set targets and offered rewards for those performing well ( Propper *et al*., 2008; Sheard, 2018: 238-9).

In some senses, ‘Targets and Terror’ succeeded. For instance, analysing English data for three common elective procedures (hip replacement, knee replacement, cataract repair) between January 1997 and December 2007, Cooper, McGuire, Jones & Le Grand (2009) found that the mean and median time patients waited between referral for admission and surgery roughly halved from their peaks to the end of the period. ^19^ Other studies found similar declines in waiting periods of six months, nine months and 12 months for a broader range of elective surgeries ( Propper *et al.*, 2008), whilst Cooper *et al*. (2009) also suggested that this and other reforms did not lead to the inequitable distribution of waiting times across socioeconomic groups that critics of the scheme predicted ( Cooper *et al.*, 2009: 675). Even scholars who highlighted managerial manipulations of targets were invested enough to recommend improvements ( Bevan & Hood, 2006). Although the scheme was scrapped after 2005, the ‘target’, as its principal technology for governing a public health system, remained.

Of course, social science and social policy scholarships were hardly unanimous in their appraisals. Academics denounced the system’s reductionism and the disempowering effects of central direction on local leadership ( Gubb & Bevan, 2009) and highlighted how ‘rhetoric about patient choice’ merely covered the creation of proxy agents: ‘quasi-markets, involving choice by the purchasing agency rather than by the patient’ ( Harrison & Mort, 1998; Mold, 2011). Many initiatives became what Harrison and Mort defined as ‘social technologies’ of legitimation for NHS and social care agencies, often having nothing to do with the patient’s voice ( Harrison & Mort, 1998: 67). And where patient groups were involved, they were now defined as ‘user groups’ (or ‘single-issue activist groups’) rather than being identified as a community of citizens as a whole. New policies developed forms of a ‘consultation *industry’* (64), with ‘active management’, rather than ‘active citizenship’ ( Milewa *et al*., 1999). ^20^

Targets and Terror formed part of this governmental genealogy as, though it explicitly aimed at reducing both waiting lists and waiting times, the programme held that the most important opinions were not those of a passive ‘customer’ but of an active ‘stake-holder’ produced through ‘active management’. What fell away in its problematization of waiting were crucial discussions about the differential *meanings* of waiting for patients; what principles might underpin an equitable distribution of waiting; what a reasonable wait for healthcare might feel like and mean to people in the context of a national health service, free at the point of entry; how waiting changes historically as an embodied experience, in what ways, and for whom, and how to make sense of the waiting that is offered to us in particular historical moments, when the interplay between abandonment and care takes on particular social, cultural and political meanings. The same can be said for even broader questions about how practitioners and patients might use time and delay within practices of care, or who might be marginalized from the very possibility of waiting as a result of treatment pathways and priorities within the system.

## Recovering histories of waiting?

In light of these occlusions – discussions overlooked in much historic and historiographic literature on the health service – is it possible to recuperate histories of waiting outside of the conjoined frames of crisis and managerialism, or the technologies of time’s economy and governance? After all, waiting *is* an elusive object. Waiting only emerges as a distinct object of attention in conditions of Modernity, as the notion of what it is that we are waiting ‘for’ has receded in secular Western Europe over the last 150 years ( Salisbury & Baraitser, 2020). Indeed, many philosophies that attempt to articulate the experience of ‘just waiting’ emerged explicitly against the increasing quantification and rationalization of time that occurred in the latter part of the 19 ^th^ century ( Schweizer, 2008), alongside a wealth of modernist literary, artistic and psychoanalytic investigations into temporal experiences that break up or break into the linear sequential arrow of time ( Kern, 1983). Waiting, we could say, understood as a lacuna that pushes its way into consciousness precisely as what we cannot grasp about time passing sequentially, is a peculiarly modern experience. It makes some sense, then, that waiting should be revealed in its negative imprint, as what evades capture even as technologies for its quantification, classification and management become ever more sophisticated.

Yet, despite waiting’s elusiveness, reclaiming certain histories of waiting within the health service is not without its possibilities. Many experiences and practices of waiting might be lost through a potent mix of the archive’s hierarchies of exclusion, while the liminality of everyday waiting has rendered it unremarked outside of certain conditions. Nonetheless, there are resources that we can turn to that might allow us to tell cultural, social and phenomenological histories of waiting in the NHS, as well as providing glimpses of its intersections with care.

For instance, although there is no ‘canon’ of artistic and pop cultural considerations of the NHS to draw upon, historians such as Mathew Thomson (2022) have begun to provide broad outlines of literary, audio, televisual and cinematic works engaging with the post-war health services and to trace historical shifts in their representation. Novels such as Margaret Drabble’s *The Millstone* (1965) and Buchi Emecheta’s *Second Class Citizen* (1974) can provide scholars with examples of powerful cultural narratives about waiting in and for health services and offer portrayals of how such waiting can feel, often drawn from personal experience. They can articulate how waiting and its inequities have fed into the complexities of NHS attachment in different historical moments: waiting for doctors manifesting as at once foreboding, boring, intolerable, a welcome escape, beset with anxiety and panic, and a prompt for reflections on racial prejudice, but the ending of which also reaffirms a sense of being reassuringly held by certain welfare services. Moreover, their examination of the social formations that historically formed within the NHS’s concrete spaces can also help articulate the affective and everyday dimensions of suspended and elongated times of care. Waiting areas and hospital wards in particular are transformed into spaces where stories and experiences are shared and anxieties are held and dissolved ( Salisbury, 2020). ^21^

Similarly productive readings might emerge from critical engagement with other sources in which patients’ and practitioners’ accounts of waiting may be constructed and preserved: newspaper and magazine articles or columns, patient letters to the press or political institutions, social and academic surveys by voluntary and commercial organizations, or by sociologists, NHS and state bodies, medical professionals, or patients’ organizations, and medical journals and popular publications, as well as memoirs and personal archive papers. ^22^ Moreover, for the post-1990 period, the careful use of web archives can provide a wealth of first-hand materials from websites, blogs, message boards and other sources. ^23^

From our initial forays, waiting is once again somewhat marginal in these materials, generally appearing as part of broader discussions of public services, health and illness, or changes in medical provision. Furthermore, where waiting is raised, it is frequently in relation to its politics and management, and generally in the form of complaint ( Moore, 2022). Indeed, where discussion has been elicited through state and health service bodies, or often White and middle-class organizations like Mass Observation, scholars must be sure to read along, as well as against, the grain. As Stoler (2009) suggests, understanding the role of such materials within forms of government, and exploring the power involved in the constructions and uses of certain narratives, are important endeavours in themselves. Nonetheless, as Daisy Payling (2020) has shown in similar contexts of complaint, combining an attentiveness to the constructed nature of these sources, together with a careful reading of their embedded values and codes, can help us recuperate important accounts of waiting otherwise made marginal in the hegemonic discourses of explored here.

The archive of NHS waiting, then, is disparate and constantly in the process of formation. It requires, perhaps, more active construction on the part of the historian than that demanded by other questions about the NHS. ^24^ Given the breadth of materials and labour required, moreover, to some extent, it is an archive only made possible by recent developments in source digitization, searchability and online preservation. ^25^

As we have been outlining here, however, we could argue that the historic and historiographic conditions giving rise and proving conducive to this work have also only formed very recently. In terms of a broader historical context, we would argue that the early years of the twenty-first century have seen an intensification of certain dominant temporal narratives: apocalyptic narratives of the ‘end times’ in relation to the climate emergency; experiences of the simultaneous speeding up of social life in relation to technology and yet a stuckness or inertness of present time brought about by conditions of neoliberal economic policies that hold citizens in states of permanent work and permanent debt; and a sense that time is ‘running out’, whereby the promises of the post-war settlement have receded and the future has become foreclosed ( Baraitser, 2017). ^26^ Together, these shifts have produced a sense of inhabiting a ‘new chronic’ time ( Cazdyn, 2012) or of living in time’s ‘suspension’ ( Baraitser, 2017), affording questions of waiting new dimensions and cultural valence. Recent literature, in other words, has both responded to and serves to analyse contemporary changes in time, simultaneously manifesting and narrating historical shifts whilst also providing the basis of new avenues for historical research.

Other historiographical developments have also been important. In particular, scholars have leveraged recent NHS milestones and responded to intensifying crisis narratives and interconnected expressions of ‘love’ for the NHS, to open up explorations of the Service’s lived and cultural history. In Snow and Whitecross’s terms, much NHS historiography prior to the last few years had revealed a considerable amount about the ‘policy, politics, and economics’ of the Service, about the dynamics and impacts of consistent service reorganizations, and themes of ‘professionalism, power distribution, workforce and organization and management’ ( Snow & Whitecross, 2022: 406). ^27^ Whilst building on this foundational literature, newer work has begun to develop cultural histories of the NHS and to reposition publics and citizens in relation to its policies and operations. Through recent publications, historians have thus increasingly moved questions about the Service’s spaces, its intersection with subjectivities and experience, and its specific forms of emotional economy, affective attachment, representation and labour, to the centre of historical analysis. ^28^ Moreover, alongside opening new angles for historical scholarship, recent projects have also provided resources for the field and for historical investigation of waiting: most notably digital galleries, new modes of engaged research and enormous repositories of oral histories to supplement existing collections ( Anucha *et al.*, 2021; Crane, 2020; Snow & Whitecross, 2022). ^29^

Together, then, a burgeoning interdisciplinary literature around waiting, and the cultural history ‘turn’ in NHS historiography, provide important intellectual frameworks and resources to address hitherto silent histories of waiting in and for the NHS, to attend more ‘care-fully’ to the shadows of waiting and care we have traced here. Moving our focus to new sources and new questions enables us, for instance, to find glimpses of ideals and practices of care, with complex relationships to ingrained clinical drives to cure or recovery. In some cases, of course, cure is impossible, and medical intervention cannot forestall death. Memoirs and observations of practice, such as Berger and Mohr's seminal *A Fortunate Man* (1967 [1997]), gesture towards the ways in which repeated attendance to terminally ill patients at home and an elongation of a visit even when ‘nothing is to be done’, have acted as offers of care to carers as much as patients. ^30^ Moreover, they highlight not just the romanticism invested in such practices of ‘waiting with’ ( Salisbury & Baraitser, 2020), but also their emotional toll on doctors, and root them in specific geographies and histories.

In other instances, the deferral of usual clinical routines and trajectories has been consciously invoked, despite other courses of action being available. For example, innovations drawn from Michael and Enid Balint’s psychanalytic work with GPs ( Bar-Haim, 2018) frequently orbited around the suspension of clinical examination, reassurance, or medication. ^31^ Balintian framings connected the urge to act in the face of patient suffering to the GP’s own emotional needs, when ‘waiting to see how the symptoms develop’ might be the better course of action where mental distress or psychosomatic illness was suspected ( Balint, 1957: 230-1). The cases collected in the Balints’ work ( Balint, 1957; Balint & Norell, 1973), offer insight into how GPs sought to mobilize these practices of waiting – whom they were prescribed for, in what instances, and how they felt for the doctor – as well as detailing how their development over the post-war years reflected shifting temporal economies within primary care.

At the same time, however, mobilizing under-used sources and new frameworks can also highlight the complexities and violences of waiting, and reinsert questions of patient voice and equity into the discussion. Doctors’ prescriptions of waiting, for instance, were not always without cost or risk, especially when made for the benefit of those beyond the patient in question. Take, for instance, GPs’ injunctions for patients to wait with supposedly ‘minor’ problems in the 1950s, made as the removal of pay barriers to healthcare brought new patients and new problems into their surgeries. Delaying would, GPs suggested, save overworked doctors’ time, enable patients with more urgent needs to be prioritized, and either allow self-resolving issues to dissipate or symptoms to develop sufficiently so diagnosis was possible ( Edwards, 1955). In rare instances, however, popular magazines offer historians the chance to see how such requests sat with (predominantly middle-class) patients. For instance, in 1953 the British Medical Association’s *Family Doctor* magazine compiled a column of letters where readers responded with palpable anxiety and confusion, noting that ‘big things have small beginnings’. They were caught, in the editors’ words, in ‘the patient’s dilemma’: torn between, on the one hand, a sense of ‘care’ for the doctor and other citizens that impelled them to defer and not ‘waste the doctor’s time’, and on the other a need for ‘reassurance’ when waiting with uncertain symptoms ( Anon, 1953), something likely heightened by emergent discourses on early diagnosis ( Armstrong, 1983). Similar affective conflicts can be detected in later community surveys of NHS use, though with concern *for* the doctor substituted with other affects. In the case of one parent interviewed by NHS researchers in mid-1980s Bloomsbury, this was – notably – fear *of* the doctor and their response. ‘I am afraid the GP will get angry with us if we have to call him home to visit, unless it is really very serious’ ( Bloomsbury Community Services Unit and Community Health Council c. 1984: 14).

Surveys such as this can feed into important social histories of waiting, underlining how health service cultures and transformation in their temporal logistics could also create racialized disparities of delay. Patients from the Chinese communities interviewed in Bloomsbury, for instance, noted how GP opening hours rarely aligned with the shift patterns and late-night work of poorer and marginalized communities; how the demands of appointments systems militated against the availability of family or friends who could act as translators; how an absence of linguistic and cultural diversity within NHS staffing made effective communication difficult and deterred attendance; and how a resulting reliance on the NHS only when patients were ‘desperate’ contributed to the development of alternative economies of private provision among those who felt excluded (14). Though echoing some common complaints made against GPs’ availability, that is, they also made plain the way in which waiting within a universal service continued to assume inequalities organized along intersections of class and race.

By embracing the research opportunities around waiting made possible by new scholarly turns, then, we can recuperate important aspects of waiting hitherto marginalized in dominant accounts of the NHS. We can also engage with broader social and health service histories, not least those that deconstruct rosier fantasies of the NHS as a haven of multi-racial harmony ( Bivins, 2017; Hunter, 2016). Furthermore, in doing so, this work might contribute to shifting contemporary reflections on and discussions of NHS waiting. It can help, for instance, articulate the potential importance of holding open time in the absence of curative possibilities ( Davies, 2022), or how waiting in uncertainty might offer more effective responses than a rush to judgment. At the same time, it can enable us to acknowledge the complexities of waiting; its potential not only for care but its contributions to – and entanglements with – forms of emotional harm and structural violence ( Baraitser & Salisbury, 2020). Even here, however, what we hope to have shown is that a relentless drive to reduce waiting *per se* is not always the most reasonable response. Instead, we need careful attention paid to the root causes of inequity and recognition of patient voices in navigating the complex and conflicting temporalities of medicine and public policy.

There is, of course, much work to be done to address the gaps currently structuring understandings of waiting in and for healthcare, both historically and today. By reviewing the state of current NHS historiography, we hope to have slowed down long enough to notice its occlusions. And together with reflections on the possibilities of future work, we hope this move has also enabled a small but significant first step towards recuperating what has been hitherto lost. Given the dangers that can emerge from a blinkered rush to action, perhaps small and deliberative steps are an essential form of care here, too.

## Data Availability

No data are associated with this article

## Footnotes

^1^ For example: https://www.independent.co.uk/news/health/nhs-70-seventy-national-health-service-video-crisis-a8432346.html; https://www.dailymail.co.uk/health/article-6491697/NHS-crisis-worse-years-worst-winter.html; https://www.theguardian.com/healthcare-network/2018/jan/04/nhs-under-threat-new-model-of-care.

^2^ For a more in-depth analysis of the crisis in the NHS see Blythe & Ross, 2022. Also: https://www.kingsfund.org.uk/publications/funding-boost-nhs-england; Emmerson & Pope, 2018.

^3^ We are grateful to the *Waiting Times* project research team – Laura Salisbury, Kelechi Anucha, Michael J. Flexer, Jordan Osserman, Stephanie Davies, and Jocelyn Catty – for the development of these ideas. *Waiting Times* is a Wellcome Trust funded research project investigating what it means to wait in and for healthcare. The project examines lived experiences, representations and histories of delayed and impeded time, and their relation to care, particularly healthcare.

^4^ Difficult, though not necessarily impossible. See, for instance: Baraitser & Brook, 2021; Day, 2015; Johnson *et al*., 2014; and especially Davies, 2022.

^5^ Similarly, see critical sociological investigations of the 1990s: Pope, 1991; Hughes & Griffiths, 1996; Hughes & Griffiths, 1997.

^6^ For discussions about the investigation of silence in the archive see: Derrida, 1996; Steedman, 2014.

^7^ See, however, some exceptions: Armstrong, 1985; Fotaki, 2006; Horobin & McIntosh, 1983.

^8^See: Adam, 1994; Adam, 1995; Adam, 2006; Appadurai, 1988; Pschetz *et al.*, 2022; Sharma, 2014.

^9^See: Chakrabarty, 2008; Edelman, 2004; Freeman, 2010; Kafer, 2013; Kristeva, 1981; Rifkin, 2017; Sharma, 2014.

^10^ See: Arney & Bergen, 1983; Horobin & McIntosh, 1983; Zerubavel, 1979. For more recent articulations examining NHS ‘crises’ see: Baraitser & Brook 2021; Wright 2022.

^11^ The Guillebaud enquiry into NHS costs was established by the Conservative Minister of Health, Iain MacLeod, in 1952. Through the work of social policy academics Richard Titmuss and Brian Abel-Smith, the committee found the NHS to be relatively underfunded and that it represented considerable value for money. See also: Sheard, 2013.

^12^ See: Long, 2013, for similar tendencies in the post-war mental health system.

^13^ Some chronically sick elderly people were referred to mental hospitals due to some confused definitions and bed shortages. See: Bridgen, 2001: 515.

^14^ In 1956, pay beds remained empty more than 50 per cent of the time. See: Williamson, 2015: 585.

^15^ The average hours worked by GPs even increased from 40 hours per week in 1965, to 42 hours, plus 23 hours on call in 1990. See: Bosanquet & Salisbury, 1998: 57–58.

^16^ See, for instance, the Patients’ Association papers at: Wellcome Library Archives, SA/PAT/D/13.

^17^ See Goddard *et al.*, 1995; Gubb & Bevan, 2009; Madhok, 1994; Martin & Smith, 1996; McAvinchey & Yannopoulos, 1993; Nikolova *et al.*, 2015; Propper *et al.*, 2002; Propper, 1988; Propper, 1990; Siciliani, 2006; Smith, 1994; van Ackere & Smith, 1999; Waller, 1996.

^18^ On NPM, see Dunleavy & Hood, 1994; Kirkpatrick *et al.*, 2005.

^19^ Mean and median times for knee replacement fell from almost 300 days in the early 2000s to under 150 days by 2007, hip replacement from 250 days to around 130 days, and cataract repair from c. 225 days in 1998 to c. 75 days in 2006. Data from Figure 1.

^20^‘Some people hold a ‘naïve’ idea that ‘active’ politics of the community is any kind of panacea for the waiting lists, implicit and explicit rationing, and prioritisation that is evident within the British health service’ ( Milewa *et al*., 1999: 461). For other critiques of active citizenship policies see: Pickard, 1998.

^21^ On *Waiting Times*, this literary archive of waiting is being explored by Kelechi Anucha in relation to end-of-life narratives and Laura Salisbury in relation to the post-war British Welfare State.

^22^ See below for citations and discussions of some of this material. But see also: Barber, 1973; Byrne & Long, 1976; Cartwright, 1967; Cartwright & Anderson, 1981; Mass Observation, 1949; Stimson & Webb, 1975.

^23^ For an innovative use of these materials in relation to the UK health services see: Millward, 2019.

^24^ Compounding the processes already involved in constructing the archive. See: Mbembe, 2002; Schwartz & Cook, 2002.

^25^ On the technical and epistemic problems raised by different types of digital sources see: Brügger & Milligan, 2019; Putnam, 2016.

^26^ For further scholarship on twenty first century temporal narratives see: Auyero, 2012; Bear, 2014; Crary, 2013; Hage, 2009; Hassan & Purser, 2007; Jeffrey, 2010; Rosa, 2013; Sharma, 2014; Southwood, 2011; Wajcman, 2015.

^27^ Indeed, some of our own scholarship has been deeply engaged in these issues. See Moore, 2019.

^28^ See Bates, 2021; Bivins, 2017; Crane & Hand, 2022; DeVane, 2021; Elizabeth *et al.*, 2019; Saunders, 2019; Simpson, 2018; Snow & Whitecross, 2022 and the wonderful work at the University of Essex: BodySelfFamily.org.

^29^ For example, collections housed at the British Library and University of Strathclyde: Bevan, 2000; Collins, 2021.

^30^ We are grateful to Laura Salisbury for her thoughts about Berger’s *A Fortunate Man.*

^31^ MM is grateful to discussions with Dr Stephanie Davies about Balintian practice and owes this framing of suspension to her.
