# Supplementary material for: The shadows of waiting and care: on discourses of waiting in the history of the British National Health Service
Source: Wellcome Open Res. 2023 Feb 13;8:73. [Version 1] doi: 10.12688/wellcomeopenres.18913.1 (PMC9978246; doi:10.12688/wellcomeopenres.18913.1)
Supplement: Peer review history

# Peer review: Reviewer response for version 1

**DOI:** 10.21956/wellcomeopenres.20970.r54949

**Published:** 2023-04-19

**Authors:** Rhodri Hayward

**Affiliations:** 1 Queen Mary University of London, London, UK

‘Time is a great healer’ is a familiar and sometimes comforting platitude but it rings hollow after an hour waiting to get through to a GPs surgery. Time is no longer seen as therapeutic or a panacea, but is felt as an irritant: an irritant which both provokes and indexes the depths of our frustration. Indeed as Bar-Haim, Baraitser and Moore demonstrate, time has become the medium through which we imagine and cost our relationship to modern health-care systems. In everyday conversations, news reports and political debates, time and time waiting, rather than mortality and morbidity, have increasingly become the measures through which most commentators assess the efficacy and success of the National Health Service. The use of such metrics now seems entirely unremarkable, but as the authors of this article make clear, the self-evidence of this approach relies upon a peculiar configuration of policies, technologies and people that has emerged in the welfare states of late twentieth-century Europe.

 This paper offers a timely intervention: the pun is not intended, but is perhaps inevitable. Over the last four decades there has been a marked and growing interest in the changing experience of time and and the roots of these different experiences.  Sociologists, geographers and historians, from Michael Young and Stephen Kern to Dipesh Chakrabarty and Rita Felski, have all urged that closer attention be paid to the tensions between different understandings and apprehensions of time. This paper - and the larger Wellcome funded project from which it stems - answer this challenge, exploring the tensions between the managerial framing of time as a metric of service provision and the ‘phenomenological temporal experiences’ of waiting and care. As the authors acknowledge, previous studies of health care and waiting lists “have, by necessity, fixed their analytic gaze upon particular political expressions of temporal suspension and elongation”, whereas their project aims to “set the ground for developing alternative historical sensibilities and perspectives: ones that might enable future work to look beyond ingrained constructions of waiting within narratives of crisis and service management.” This is a laudable aim. It is therefore all the more regrettable, that despite the brilliance of the research and analysis here, the opportunity to interrogate personal experience is never taken. Faced with the obscurity of individual experiences of waiting, the authors set out instead to trace ‘its more visible twin’, the health service policies that have sought to manage the timings of treatment and the provisions of care.

 In pursuit of the ‘more visible twin’ paper lays the groundwork for a future analysis of waiting. It sensibly limits itself to a reconstruction of the ways in which waiting became a marker of service provision. This reconstruction is pursued through a series of ‘snapshots’ of: policy changes, namely: 1) the NHS Bed Crisis of the late 1940s and 1950s which in the wake of the 1956 Guillebaud Committee led to a series of managerial reforms; 2) the rise of primary care appointment systems that followed the negotiations around 1966 GP contract; and 3) the neoliberal turn to consumerist and managerial systems of health care epitomised in the 1983 Griffiths Report. These snapshot studies of policy shifts provide brief but illuminating examples of the ways in which experiences of waiting were turned into objects of political discussion and measures of managerial efficacy. The ‘snapshot’ is a curious choice of metaphor for organising and presenting changing experiences of time granted that the photograph, as a host of academic commentators have recognised, embodies a particular temporality: a time that is frozen, past and torn from its context. The snapshot method, then, produces a series of vignettes which are by necessity insulated from other contemporaneous developments in other parts of the health service and from broader historical shifts in the experience and understanding of time. 

 The importance of moving beyond the snapshot partly lies in the fact that different medical specialisms maintain different investments in time as becomes clear in a comparison of specialist and primary care. Throughout the twentieth-century the professional identity of general practitioners was bound up with their claims to a unique position as ongoing witnesses to their patients’ lives. Similarly, the changing understandings of diseases and their development produced in clinical research cannot help but inform or accentuate both the patient and the family doctor’s understanding of waiting. It is difficult to maintain the insularity that the snapshot form demands while hoping to trace out the shadow or obscure shape of a patient’s experience. It sometimes feels like we are looking the wrong way down a telescope. Had we started with the experiences of patients, for instance W. N. Barbellion’s (G. F. Cummings) Edwardian, memoir of multiple sclerosis, one would quickly apprehend the multiple temporalities folded into a single case of waiting, as memories of the cases of families and friends, political loyalties or the demands of work inform the experience of a situation. Likewise turning to the close study of a practitioner’s experience (such as when the authors gesture towards John Berger’s study of the Gloucestershire GP, John Sasssall (pseud. John Eskell) in *A Fortunate Man *(1964)), reveals the ways that time and waiting shift over the course of a life and in the midst of an illness.

 The authors of this paper are aware of this, but it also illustrates the sheer difficulties of their stated task. The paper does offer a deeply informed and theoretically sophisticated analysis of the emergence of time as an object and metric in health service management, and its deftly shows how this has worked to reshape political debate. Yet it remains difficult to see how much this can help us recover more than the haziest ‘shadow of waiting’ as an experience. What the paper provides is a clear sense of the political aims and economic constraints that have combined to elide waiting and frustration. In making clear the specific political and material forces that have created this situation, it reminds us that other kinds of waiting are possible. One is left wondering what kind of technological or social breakthrough would be needed to transform waiting on a GP surgery phone line from a form of frustration to a new sense of hopeful anticipation.

Is the work clearly and accurately presented and does it cite the current literature?

Yes

If applicable, is the statistical analysis and its interpretation appropriate?

Not applicable

Are all the source data underlying the results available to ensure full reproducibility?

No source data required

Is the study design appropriate and is the work technically sound?

Yes

Are the conclusions drawn adequately supported by the results?

Yes

Are sufficient details of methods and analysis provided to allow replication by others?

Yes

Reviewer Expertise:

Modern British history

I confirm that I have read this submission and believe that I have an appropriate level of expertise to confirm that it is of an acceptable scientific standard.

# Peer review: Reviewer response for version 1

**DOI:** 10.21956/wellcomeopenres.20970.r54950

**Published:** 2023-03-01

**Authors:** Alex Mold

**Affiliations:** 1 Centre for History in Public Health, London School of Hygiene and Tropical Medicine, London, UK

This is a rich and engaging piece of work that explores new ground within the history and historiography of the National Health Service in Britain. The article traces the development of discourses around waiting in the NHS since the 1950s. It charts a narrowing of the understanding and representation of ‘waiting’ to waiting lists and wait times, even as waiting as an experience remained diverse and multi-faceted. The authors point out that waiting is now often seen as a proxy for the health or otherwise of the NHS, but waiting had and has a set of broader functions and meanings that this article sets out to recover. This is especially true of the role of waiting in care and treatment, which, the authors suggest, needs better conceptualisation. 

 The authors argue that the history of waiting has something important to contribute to contemporary analysis: by recovering the different functions and trajectories of waiting nuance can be added. There was an opportunity here to have been bolder about what exactly this more nuanced understanding of waiting would lead to. There is an implication, for instance, that a deeper understanding of waiting might involve more patient voice, or greater efforts to improve equity, rather than simply focusing on waiting lists and times. The authors might also have considered the possibility that a more ‘positive’ account of the value of waiting could contribute to contemporary justification for waiting, possibly to the detriment of some patients. 

 The authors locate their work within an emerging historiography of the NHS that moves beyond systems, policies, and politics to explore the deeper social and cultural meanings attached to healthcare in the UK. This article will form an important part of this literature, both in terms of its content, but also in relation to its methodology, which looks for the hidden narratives around waiting as well as the explicit. The article is bold and convincing in its approach here and will set the direction for many future pieces of work.

 The main body of the article examines discourses on waiting in the historical, sociological, and economic literatures on the NHS at three key moments: the 1950s bed crisis; general practice in the 1960s; and managerial culture in the 1980s-90s. These are all important moments, but the authors could have been more explicit about how and why they decided to focus on these case studies. Would different moments tell a different story? There is inevitably some simplification necessary in recounting these episodes, and this results in some loss of detail. Is it right, for instance, to characterise the 1950s-70s debates about beds as a ‘crisis’? As the authors point out, this went on for a long time and took various forms that were not just about responding to a ‘crisis’. The authors also suggest that during the 1980s waiting was reformulated as being primarily about inefficiency. It was not totally the case that concerns about inequity also disappeared though, as there was plenty of attention paid to this not only by patient groups (which the authors acknowledge) but also from the media. This was especially the case, for instance, around ‘postcode lotteries’ in accessing care and wait times. 

 Beyond the case studies, the article presents an analysis not just of narratives of waiting, but also the place of time within healthcare more widely. In places there was a slippage between time and waiting, and some more specificity might have added to the analysis. For instance, the discussion around the quantification of time within general practice could have linked back more explicitly to waiting – after all the introduction of appointment systems within general practice did not get rid of waiting entirely, but perhaps it did change patients’ experience and perception of this?

 Although this may well be beyond the scope of this article, it might also be worth reflecting on the NHS-ness of these discourses. How specific is this framing of waiting to the UK? Waiting for and in healthcare is not unique to Britain, but presumably there are elements that are specific to the NHS, and others that are more universal. 

 Overall, these points do not detract from a tremendously engaging and important piece that will be widely read and referenced for years to come.

Is the work clearly and accurately presented and does it cite the current literature?

Yes

If applicable, is the statistical analysis and its interpretation appropriate?

Not applicable

Are all the source data underlying the results available to ensure full reproducibility?

No source data required

Is the study design appropriate and is the work technically sound?

Yes

Are the conclusions drawn adequately supported by the results?

Yes

Are sufficient details of methods and analysis provided to allow replication by others?

Yes

Reviewer Expertise:

History of public health

I confirm that I have read this submission and believe that I have an appropriate level of expertise to confirm that it is of an acceptable scientific standard.

# Peer review: Reviewer response for version 1

**DOI:** 10.21956/wellcomeopenres.20970.r54948

**Published:** 2023-02-20

**Authors:** David Armstrong

**Affiliations:** 1 Department of Primary Care and Public Health Sciences, King’s College London, London, UK

This paper provides an overview of the concept of ‘waiting’ during the history of the British NHS.

 Overall, I thought this was an interesting paper that identified an approach, the idea of ‘waiting’, that enables an original slice through different aspects of the NHS since its inception. Full marks for identifying this rich analytic theme. The paper is fairly schematic and lays out the broad landscape for future investigations. But in showing the potential for this sort of analysis it should elicit wider interest.

 I recall many American students of the NHS were impressed by many of its features but were always critical of waiting lists which seemed its constant downside. My response, building on some basic economics, was that where there is more demand than supply there has to be a rationing mechanism. In the US that was largely financial – if you couldn't afford healthcare then you never joined the waiting list – but in the absence of financial barriers other impediments emerged in the NHS. One of these was ‘inconvenience’: if a service was sufficiently inconvenient to use then demand would be reduced. As this paper makes clear, one of the primary elements of that inconvenience was waiting and how waiting was conceptualised, operationalised, prioritised, etc, provides a novel framework for analysing the history of the NHS.

 The paper also begins to address the problem of time itself, which underpins much of the waiting time/list debate. There are some nice touches (eg ‘Time is framed as a socially produced narrative, or as a category of experience that itself has a ‘social life’ disrupting the idea that time is a blank backdrop against which history unfolds’) which indicates the sorts of reflexivity that will be needed for further pursuit of this project. But this is a good start.

 The only section that I thought could perhaps have been explored in a little more detail was around the ‘shortage of beds’. The phenomenon of fewer beds was not only a ‘cause’ of longer waiting lists but also a consequence of changes in waiting patterns. For the early NHS years patients could spend weeks in hospital ‘recuperating’ or 'convalescing' but then in the 1960s a discourse on the dangers of being bed-bound emerged and early mobilisation and discharge became therapeutic goals. In effect, the time ‘waiting’ to go home was reduced meaning that fewer beds were needed. This phenomenon is particularly stark in the massive decline in psychiatric beds – another example of patients waiting, often indefinitely, but then being discharged. In fact, for most of its history the NHS has been closing beds as community care, day case or out-patient management, etc, reduced their need. Though of course how these changes were interpreted in terms of the waiting time/list discourse is another matter.

Is the work clearly and accurately presented and does it cite the current literature?

Yes

If applicable, is the statistical analysis and its interpretation appropriate?

Not applicable

Are all the source data underlying the results available to ensure full reproducibility?

No source data required

Is the study design appropriate and is the work technically sound?

Yes

Are the conclusions drawn adequately supported by the results?

Yes

Are sufficient details of methods and analysis provided to allow replication by others?

Yes

Reviewer Expertise:

sociology of medical knowledge, health services research

I confirm that I have read this submission and believe that I have an appropriate level of expertise to confirm that it is of an acceptable scientific standard.
